# Single-step genomic BLUP enables joint analysis of disconnected breeding programs: an example with *Eucalyptus globulus* Labill

**DOI:** 10.1093/g3journal/jkab253

**Published:** 2021-07-16

**Authors:** Andrew N Callister, Ben P Bradshaw, Stephen Elms, Ross A W Gillies, Joanna M Sasse, Jeremy T Brawner

**Affiliations:** 1 Treehouse Forest Research LLC, Check, VA 24072, USA; 2 Australian Bluegum Plantations, Albany, WA 6330, Australia; 3 HVP Plantations, Churchill, VIC 3842, Australia; 4 Sassafras Group Pty Ltd, Yarraville, VIC 3013, Australia; 5 Plant Pathology, University of Florida, Gainesville, FL 32611, USA

**Keywords:** breeding value accuracy, forest tree breeding, genomic relationship matrix, genomic selection, HBLUP, Myrtaceae, MPP, Multiparental Populations

## Abstract

Single-step GBLUP (HBLUP) efficiently combines genomic, pedigree, and phenotypic information for holistic genetic analyses of disjunct breeding populations. We combined data from two independent multigenerational *Eucalyptus globulus* breeding populations to provide direct comparisons across the programs and indirect predictions in environments where pedigreed families had not been evaluated. Despite few known pedigree connections between the programs, genomic relationships provided the connectivity required to create a unified relationship matrix, **H**, which was used to compare pedigree-based and HBLUP models. Stem volume data from 48 sites spread across three regions of southern Australia and wood quality data across 20 sites provided comparisons of model accuracy. Genotyping proved valuable for correcting pedigree errors and HBLUP more precisely defines relationships within and among populations, with relationships among the genotyped individuals used to connect the pedigrees of the two programs. Cryptic relationships among the native range populations provided evidence of population structure and evidence of the origin of landrace populations. HBLUP across programs improved the prediction accuracy of parents and genotyped individuals and enabled breeding value predictions to be directly compared and inferred in regions where little to no testing has been undertaken. The impact of incorporating genetic groups in the estimation of **H** will further align traditional genetic evaluation pipelines with approaches that incorporate marker-derived relationships into prediction models.

## Introduction

Quantitative genetics has utilized pedigree-derived relationships to improve selection efficiencies of breeding programs for many years (Henderson 1975). Well-defined relationships within the populations improve the accuracy of breeding value estimates, the partitioning of genetic variance, and the estimation of environmental effects. Forest tree breeding has delivered greater gains over time as the relationships amongst experimental units have increased in precision from open-pollinated (OP) families to control-pollinated (CP) families (McKeand and Bridgwater 1998), to progeny themselves (“individual model”; Borralho 1995), to clonally replicated progeny (Costa e Silva *et al.* 2004; Isik *et al.* 2005; Callister and Collins 2008; Baltunis *et al.* 2009).

Genetic markers such as single nucleotide polymorphisms (SNP) provide empirical rather than expected relationships by using estimates of allele similarity between pairs of individuals (VanRaden 2008). Genotyping provides direct estimates of relationship coefficients that may replace or be combined with expected values derived from the documented pedigree. Relationships determined by genotyping have been used to recover nonadditive genetic variances from OP tree families (Klápště *et al.* 2014; Gamal El-Dien *et al.* 2016) and remove inflation in half-sib-based additive genetic variance estimates (Klápště *et al.* 2014; Ratcliffe *et al.* 2017). Large-scale genotyping in OP families has also produced substantial improvements to breeding value accuracy (Gamal El-Dien *et al.* 2016; Thavamanikumar *et al.* 2020) and accounted for inbreeding depression (Klápště *et al.* 2017, 2018). In hybrid tree populations, genotypic relationships have revealed the magnitude of epistatic variation and partitioned genetic variances more precisely (Bouvet *et al.* 2016; Tan *et al.* 2018). Genotyping also resolved errors in pedigree information and improved model fit in a clonally replicated full-sib population of loblolly pine (Munoz *et al.* 2014).

Although genotyping has demonstrated great promise for improving relationship estimation, industrial breeding programs are comprised of thousands of individuals in multigenerational populations and genotyping entire populations is not possible or impractical. Animal breeders faced with a similar challenge developed the “single-step genomic BLUP” procedure, in which empirical relationship estimates from a genotyped subset (**G**) are merged into the traditional pedigree-derived matrix of expected relationship coefficients (**A**) of the entire population (Legarra *et al.* 2009; Misztal *et al.* 2009; Christensen and Lund 2010). The resulting relationship matrix (**H**) is then used in place of **A** in linear mixed models (LMM) to produce Best Linear Unbiased Predictions (BLUP) of genetic value. This “HBLUP” approach has been combined with efficient computing procedures (Misztal *et al.* 2009) to allow for genetic evaluations that integrate hundreds of thousands of genotypes with phenotypes from millions of animals (Misztal *et al.* 2013a). The approach is regularly used for genomic evaluation and selection in many livestock species (Lourenco *et al.* 2020).

HBLUP has been applied to the analysis of growth and wood quality traits for OP populations of *Picea glauca* (Ratcliffe *et al.* 2017), *Eucalyptus grandis* (Cappa *et al.* 2017; 2018), *Eucalyptus nitens* (Klápště *et al.* 2018), and *Eucalyptus pellita* (Thavamanikumar *et al.* 2020) and for OP and CP families of *Pinus contorta* (Ukrainetz and Mansfield 2020). These studies involved genotyping of progeny and unanimously revealed the presence of relationships amongst parents that had not been previously known. Assumed relationships between parents and progeny (expected 0.5) and between half-sib progeny (expected 0.25) are more precisely estimated when blended with genomic similarities in **G** to create the **H** matrix (Cappa *et al.* 2017). A greater density of relationship information in **H** compared with the sparse relationships in **A** led to improved theoretical accuracy of estimated breeding values (EBVs), in some cases only for genotyped individuals (Ratcliffe *et al.* 2017) and in other cases for ungenotyped individuals and parents as well (Cappa *et al.* 2017; Thavamanikumar *et al.* 2020). Phenotypic information from trees that have not been genotyped was also shown to improve genomic predictions (Cappa *et al.* 2019). In forest tree populations, HBLUP efficiently combines genomic, pedigree, and phenotypic information and offers substantial advantages over conventional pedigree-based analysis. On the other hand, the published studies applying HBLUP to forest tree populations have all been limited to small experimental populations, ranging upwards to 5742 genotyped and ungenotyped progeny (Thavamanikumar *et al.* 2020). This is one to two orders of magnitude smaller than required for long-running commercial tree improvement programs. Ukrainetz and Mansfield (2020) noted computational limitations extending their methodology to larger phenotypic and marker datasets while recognizing that HBLUP has been applied to millions of individuals in animal breeding applications (*e.g.*, Tsuruta *et al.* 2021). Scaling up the HBLUP approach to a complete multigenerational breeding population is required to integrate this technology into applied tree breeding programs.

*Eucalyptus globulus* Labill. is a commercially important plantation species in Mediterranean climates around the world and is particularly favored for high-yielding short-fiber pulpwood crops. Approximately 460,000 hectares of *E. globulus* plantations are managed in Australia (Downham and Gavran 2019), principally in three major planting regions: (1) Southwest Australia (50% of area), (2) the “Green Triangle” of western Victoria and southeast South Australia (32% of area), and (3) central and eastern Victoria (10% of area). The species’ native range in Southeast Australia has been classified into 13 races and 20 subraces (Dutkowski and Potts 1999; Potts *et al.* 2004). Landraces are also recognized where *E. globulus* has been naturalized in areas such as Portugal, Spain, and Chile. Racial variation in evolutionary and commercial traits has been well described (Lopez *et al.* 2001). Narrow-sense heritability estimates from *E. globulus* progeny trials are generally low for growth and moderate to high for wood basic density and pulp yield (Costa e Silva *et al.* 2009; Callister *et al.* 2011; Araujo *et al.* 2012). Nonadditive genetic effects on *E. globulus* growth can be significant (Araujo *et al.* 2012). Genotype–environment interaction has been reported for *E. globulus* at the regional scale across Australia. Costa e Silva *et al.* (2006) reported an average intersite correlations amongst four Australian growing regions of 0.73 for subrace effects and 0.76 for OP family effects and regional correlations are accommodated in routine analyses of Australian breeding populations (*e.g.*, Dutkowski *et al.* 2015).

Most commercial tree improvement activities are practiced within the confines of an individual program. However, important exchanges of information and genetic material also occur amongst programs to support breeding and the establishment of planted forests. A major limitation to such exchanges exists when pedigrees have been coded differently and little connectivity is available between programs to provide unbiased comparisons among populations. This situation provided the stimulus for our work to quantify the impact that combining two distinct pedigrees into a joint relationship matrix (**H**) has on genetic parameter estimates and the accuracy of breeding value predictions. Replacement of **A** with **H** as the numerator relationship matrix (NRM) involves two types of changes to the information used in the analysis. Not only do genomic data inform the relationships amongst individuals in **H**, but the formulation of **H** applied thus far in tree breeding does not allow for races as genetic groups. Therefore, we aimed to disentangle the impact of including genomic information from that of removing race groups by comparing results from three types of models: ABLUP with race genetic groups (ABLUP_+race_; benchmark model), ABLUP without groups (ABLUP_−race_), and HBLUP.

A second motivation was to demonstrate a methodology for combining large datasets in anticipation of a new generation of analytical tools. The newly emerging ability to model genetic responses to environmental attributes presented as continuous surfaces (Resende *et al.* 2020), to predict unobserved phenotypes through environmental relationship matrices (Jarquín *et al.* 2014), and to synthesize large ‘omics datasets into coherent models of plant function (Weighill *et al.* 2019) rely on very large experimental populations distributed across the landscape. The most informed results from these alternative analytical approaches may be derived from datasets that include structured genetic effects, such as those managed in formal breeding programs evaluating diverse sets of germplasm across a wide range of environments.

## Materials and methods

### Experimental populations

This study was conducted using two completely separate multigenerational breeding populations of *E. globulus* in southern mainland Australia, EG1 (Australian Bluegum Plantations) and EG2 (HVP Plantations). These commercial tree improvement programs progeny test full-sib families predominantly derived from first- and second-generation selections. Forty-eight field trials established between 1998 and 2015 provided phenotypic data from 126,467 full-sib progeny (1973 families), 7051 half-sib progeny (263 families), and 24,767 unpedigreed trees of commercial entries (193 “checklots”; Table 1 and Supplementary Table S1A describes progeny trial network).

**Table 1:** Overview of genetic entries in the analyzed data

Program	Generation	Entries	Individuals
Full-sib families			
EG1	2	453	38,846
	3	816	41,775
	4	13	458
EG2	1	2	166
	2	509	35,042
	3	180	10,180
Full-sib subtotal		1,973	126,467 (80%)
Half-sib families			
EG1	1 and 2	261	6,852
EG2	1	2	199
Half-sib subtotal		263	7,051 (4%)
Checklots			
EG1	n/a	155	15,477
EG2	n/a	38	9,290
Checklots subtotal		193	24,767 (16%)
Total		2,429	158,285

**Figure 1:**
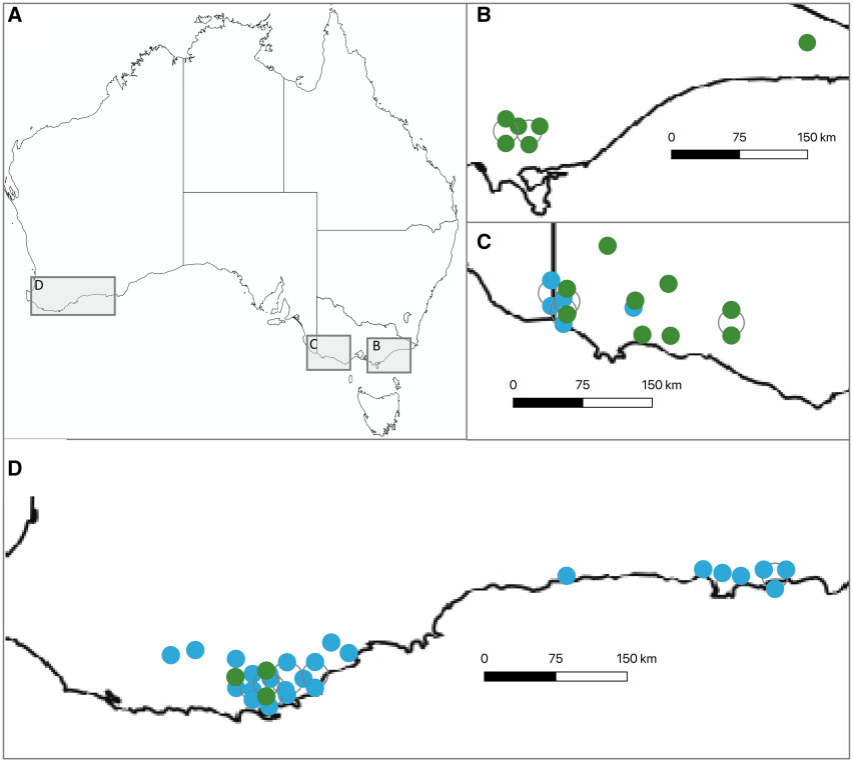
Locations of the 48 trials used for this study with (A) reference map of Australia, (B) 6 trials in Gippsland, (C) 14 trials in the Green Triangle, and (D) 28 trials in Western Australia. EG1 trials represented as blue symbols, EG2 trials as green symbols.

Field trials were located in three regions of southern Australia: Western Australia (WA), the Green Triangle (GT), and Gippsland (GIPPS). EG1’s breeding program was focused in WA (25 trials) with five trials in GT and none in GIPPS. EG2 trials were located predominantly in GT (nine trials), with six trials in GIPPS and three in WA (Figure 1, Supplementary Table S1B describes trial distribution across regions).

The EG1 population of full-sib families was founded on 112 base-population trees and the EG2 population was founded on 83 base-population trees (Supplementary Table S1C describes populations). These “founders” are trees in native forests or international landraces that provided the seed for testing and domestication. Half the founders of the EG2 program originated from Strzelecki Ranges and Western Otways races, whereas the EG1 program is more strongly influenced by Furneaux, King Island, Portugal (landrace), and NE Tasmania. The classification of races follows the results of Dutkowski and Potts (1999). A total of 347 EG1 parents and 107 EG2 parents were represented by progeny included in these analyses.

Inadequate interprogram relationships were available for joint analyses using pedigree derived relationships as only four founders were common to both programs. Pedigree-derived relationship coefficients amongst the two parent cohorts were rare, with two relationships of 0.25, 152 relationships of 0.125, and one expected relationship coefficient of 0.0625.

Trials were primarily established in randomized incomplete-block designs (two EG1 trials were alpha-cyclic incomplete row-column designs) with four to eight replications of each family established in four- to five-trees in contiguous row-plots. Two EG1 trials were single-tree plot designs. Incomplete blocks were arranged into replicates that were generally contiguous to enable the resolution of spatial trend effects within each trial.

### Phenotyping of field trials

Diameter at breast height (DBH) was measured with a tape for each tree at 3 years (7 sites), 4 years (9 sites), 5 years (29 sites), or 8 years (3 sites; see Supplementary Table S1A) after planting. Tree height (HT) was measured with a hypsometer for each tree in the majority of trials, although a subset of 3–17% (mean 10%) of trees were assessed in 11 of the EG2 trials. In these cases, unmeasured HT data were predicted from the DBH–HT relationship established amongst measured trees for each trial. Stem volume (VOL) was calculated for each tree using DBH, HT (measured or predicted), and the region-specific volume function provided by each program. DBH of all stems forking below breast height was measured in 36 trials and in these cases, VOL was aggregated to the tree level before analysis.

Given the expense of wood property assessments, a subset of trees in 20 of the 48 trials were selected for phenotyping (Supplementary Table S1D describes subset of populations assessed for wood properties). Basic density (DENS) data were collected for a subset of trees between 5 and 8 years. EG2 measured DENS directly by the displacement method using the outer 50 mm of wood cores extracted at breast height. EG1 used pilodyn penetration as an indirect measure of wood density (Muneri and Raymond 2000). Pilodyn data were converted to outer wood DENS using the equation from Callister and England (2010). Cellulose content (CELL) was predicted by Forest Quality Pty Ltd in Tasmania, Australia using near-infrared spectrometry. Samples of either whole-wood cores or shavings taken from the outer 50 mm of the stem between 5 and 8 years of age were ground and scanned to produce spectra for cellulose prediction (Downes *et al.* 2010).

### Genotyping

Leaves were sampled from 164 parents and 74 others in the EG1 program and from 93 parents and 51 others in the EG2 program, where “others” refers to phenotypic selections that have not been progeny tested. DNA extraction and genotyping were conducted at Gondwana Genomics Pty Ltd, Canberra (Thavamanikumar *et al.* 2020). The *E. globulus* marker panel consisted of 2579 SNP and small biallelic insertion/deletion (INDEL) markers within candidate genes associated with diameter, density, and cellulose yield that were identified using association analyses in previous studies (Southerton *et al.* 2011). After filtering out markers with minor allele frequency less than 5%, 2444 markers were available from 382 parents for the derivation of relationship matrices.

### Statistical analyses

#### Relationship matrices

Relationship matrices were produced using *preGSf90*, which is a module of the Fortran-based *BLUPF90* suite (Misztal *et al.* 2014). The default settings of *preGSf90* were used for quality control, imputation of missing markers, and calculation of the relationship matrices (Aguilar *et al.* 2014). The EG1 **G** matrix (**G_EG1_**) was formed for 238 genotyped individuals, the EG2 **G** matrix (**G_EG2_**) was formed for 144 genotyped individuals, and the joint **G** matrix (**G_JOINT_**) was formed amongst all 382 genotyped individuals. **G** was calculated in *preGSf90* following the first method of VanRaden (2008):

#(1) G=ZZ'2∑jpj1-pj,

where **Z** is a matrix of centered genotype scores calculated as **Z **=** M** − 2 **P**; **M** is the *n* × *m* matrix of *n* genotypes and *m* markers scored 0 for homozygous reference allele, 1 for heterozygous, and 2 homozygous alternative allele, **P** is a matrix of frequency for the alternative allele, and *p_j_* is the reference allele frequency of the *j*th marker.

The pedigree relationship matrix **A** was divided into four submatrices: **A**_11_ representing relationships amongst nongenotyped individuals, **A**_22_ representing relationships amongst genotyped individuals, and **A**_12_ and **A**_21_ representing relationships between genotyped nongenotyped individuals:

#(2) A=A11A12A21A22.

Differences in the constitution of contemporary and historical populations can lead to different mean breeding value and genetic variance between **G** and **A_22_** (Forni *et al.* 2011; Vitezica *et al.* 2011). We therefore rescaled **G** to make it compatible with **A_22_** following the approach detailed in Christensen *et al.* (2012):

#(3) Ga=a+bG,

where *a* and *b* were determined by solving the following system of equations:

#(4) a+bdiagG-=diagA22-a+bG-=A22-.

The rescaled matrix **G_a_** was then weighted to avoid difficulties with inversion (Aguilar *et al.* 2010):

#(5) Gw=0.95Ga+0.05A22,

which was then used to calculate **H** (Legarra *et al.* 2009; Christensen and Lund 2010):

#(6) H=A11-A12A22-1A21+A12A22-1GwA22-1A21A12A22-1GwGwA22-1A21Gw

or equivalently (Lourenco *et al.* 2020):

#(7) H=A11+A12A22-1(Gw-A22)A22-1A21A12A22-1GwGwA22-1A21Gw

with inverse

#(8) H-1=A-1+000Gw-1-A22-1.

**H** was then resorted to align with the pedigree relationship matrix for comparisons with **A**. The section of **H** relating to genotyped individuals, their parents, and ancestors were compared with **A** in a preliminary step to highlight identity errors and mistakes in the documented pedigree. These errors were rectified in the pedigree and the process of creating the **H**^−1^ matrix was then repeated with the correct pedigree information.

#### Genetic analyses

All genetic analyses were conducted using ASReml v. 4.1 (Gilmour *et al.* 2015). Models incorporating among individual relationship matrices derived from pedigree (ABLUP) or markers and pedigree (HBLUP) were fit following Henderson (1984) with a general LMM:

#(9) y=Xb+Zu+e,

where **y** is the vector of phenotypic values, **X** is the incidence matrix for fixed effects, **b** is the vector of fixed effects, **Z** is the incidence matrix for random effect, **u** is the vector of random effects with E(**u**) = 0, and **e** is the vector of residual effects expected to be independently normally distributed with E(**e**) = 0. The relationship matrix that differentiates ABLUP and HBLUP connects each individual in **u**, and this change in connectivity impacts genetic parameter estimates and the accuracy of breeding value predictions.

Single-site ABLUP analyses were first conducted for each trial to provide spatially adjusted volume (VOL_adj_) data that were used for preliminary cross-site analyses (following Ye and Jayawickrama 2008). Fixed effects were included for the overall mean, replicates, and checklots. Random effects included additive genetic effects, family-specific genetic effects specifying the combination of parents evaluated by control pollinated progeny, incomplete block effects, and row-plot effects. The races listed in Supplementary Table S1C were included as fixed genetic group effects within the pedigree (Westell *et al.* 1988). Genetic parameter estimates for each trial did not vary substantially from expectation and all trials were retained for across-site analyses. Preliminary cross-site analysis of VOL_adj_ provided additive variance estimates for each trial, which were used to standardize each trial to have a mean of zero and an additive standard deviation of one. Partitioning of additive and nonadditive effects depended on the structure of the families evaluated in different trials and this heterogeneity was accommodated by estimating variance four groups of trials with similar ratios of family to additive variance. A more detailed description of the single site and preliminary analyses is provided in Supplementary Methods as SM.M1 and SM.M2.

Multivariate models were fit to VOL_adj_, DENS, and CELL separately using an unstructured variance model that provided additive genetic variance estimates for each region and inter-region correlations. This model produced genetic parameter estimates as well as breeding value predictions with their associated accuracy for all trees in each region. Comparisons of genetic parameters and accuracy estimates when different models are used for different sets of the populations are detailed in Table 5, Supplementary Tables S2, A and B. Site-specific effects for incomplete blocks, plots, and multistem form were modeled as random effects specific to each individual site. OP family means were fitted separately and did not contribute to estimates of ${\hat{\sigma }}_{a}^{2}$. Checklots were fitted as fixed effects and additional within-plot error was fitted to checklots at each site separately. The cross-site modeling progressed for each program using **A** with genetic groups, **A** without groups, and **H** without genetic groups as the NRM. Finally, the joint **H** was used with data from both programs in combined analyses.

Narrow-sense heritability and dominance proportions for VOL were estimated at regional scales as follows:

#(13) h^regional2=σ^aregioni2σ^aregioni2+σ^fregioni2+∑k=1nσ^bk2+σ^pk2+σ^ek2n

#(14) d^regional2=4σ^fregioni2σ^aregioni2+σ^fregioni2+∑k=1nσ^bk2+σ^pk2+σ^ek2n,

where ${\hat{\sigma }}_{a_{{\mathrm{region}}_{i}}}^{2}$ is the cross-site additive genetic variance estimate for the *i*th region, ${\hat{\sigma }}_{f_{{\mathrm{region}}_{i}}}^{2}$ is the cross-site family-specific variance estimate for *i*th region, and $\sum_{k=1}^{n}\left({\hat{\sigma }}_{b_{k}}^{2}+{\hat{\sigma }}_{p_{k}}^{2}+{\hat{\sigma }}_{e_{k}}^{2}\right)$ is the sum of variance due to incomplete blocks, plots, and residuals used to estimate the mean across *n* trials in each region. Region-specific estimates of variance components were used to estimate ${\hat{h}}_{\mathrm{regional}}^{2}$ and ${\hat{d}}_{\mathrm{regional}}^{2}$ separately for VOL in WA, GT, and GIPPS. Trials contributing to the estimate of family variance were identified as the Group 2 trials in Supplementary Methods SM.M2.

Narrow-sense heritability and dominance proportions for DENS and CELL were estimated across all sites basis as follows:

#(15) h^Xsite2=σ^a2σ^a2+σ^f2+∑k=1nσ^bk2+σ^pk2+σ^ek2n

#(16) d^Xsite2=4σ^f2σ^a2+σ^f2+∑k=1nσ^bk2+σ^pk2+σ^ek2n,

where ${\hat{\sigma }}_{a}^{2}$ is estimated across sites, ${\hat{\sigma }}_{f}^{2}$ is the family variance approximated across groups of trials with different ${\hat{\sigma }}_{f}^{2}:{\hat{\sigma }}_{a}^{2}$ ratios as described in Supplementary Methods SM.M2, so that ${\hat{\sigma }}_{f}^{2}$ is a weighted mean estimate of family variance across all *n* sites. Other terms are defined as above for VOL.

Following the recommendation of Putz *et al.* (2018), the accuracy of breeding value predictions was used to provide empirical comparisons of the accuracy of traditional and HBLUP models for different sets of the population. The accuracy (${\hat{r}}_{g\hat{g}}$) of predictions was calculated as:

#(17) r^gg^=1-PEVσa2.

PEV estimates for each individual are derived from the inverse of the relationship coefficient matrix. The method for PEV approximation in ASReml is described by Welham *et al.* (2004) and Gilmour *et al.* (2004), which utilize methods suggested by Misztal and Wiggans (1998) and Kackar and Harville (1984) to approximate prediction errors as suggested by Henderson (1976).

## Results

### Relationship matrices

**Figure 2:**
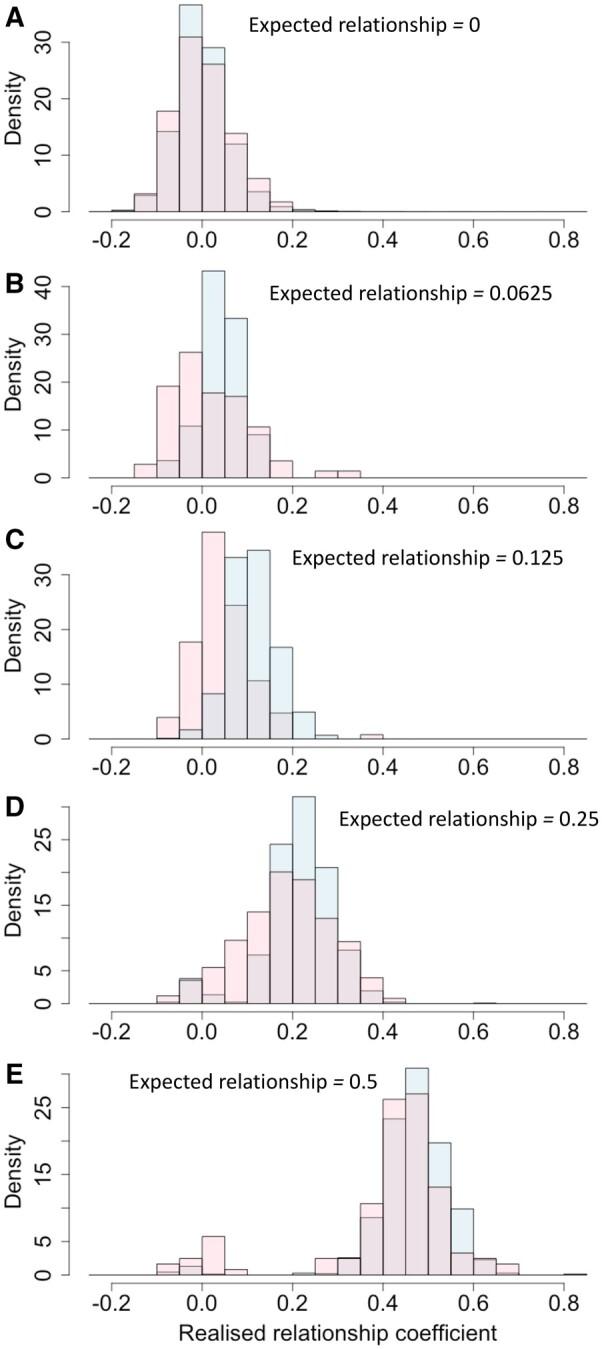
Density distribution of realized relationship coefficients within the **G** matrix of the EG1 program (blue bars) and EG2 program (pink bars) for relationships with expected coefficient of (A) zero, (B) 0.062, (C) 0.125, (D) 0.25, and (E) 0.5.

Realized relationship coefficients amongst genotyped individuals in **G_EG1_** and **G_EG2_** were distributed around values expected from pedigree relationships (Figure 2). Note that these relationship matrices contain covariances between individuals estimating similarities in allele frequencies rather than probability estimates of shared identity by descent derived from pedigree. A small number of realized relationship coefficients that diverged excessively from expected values, highlighting individuals with erroneous pedigree information, were corrected by reassigning parentage before further analyses.

**Figure 3:**
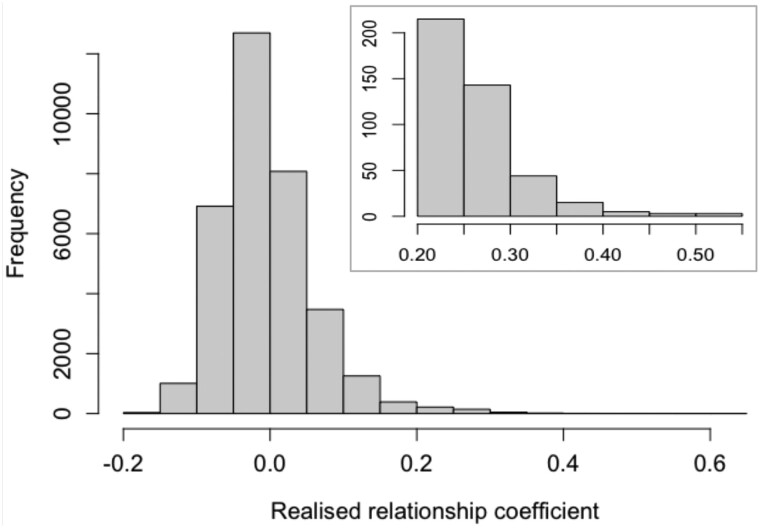
Frequency of relationship coefficients between programs in the joint **G** matrix. Inset provides detail for 428 relationships with *r* > 0.2

**G_JOINT_** revealed unknown relationships amongst parents in the experimentally disconnected breeding programs. Although the majority of the 34,272 interprogram relationships in **G_JOINT_** were near zero, 2071 relationship coefficients were greater than 0.1, 428 were greater than 0.2, and 70 were greater than 0.3, with a maximum of 0.54 (Figure 3). Using genomic rather than pedigree-derived relationships provided sufficient cross-program linkage within **G_JOINT_** to proceed with forming **H_JOINT_**.

Relationships that were quantified in **G** were projected into **H** to reveal relationships assumed to be zero in **A**. This was observed within relationship matrices for EG1 and EG2 separately and jointly to provide comparisons of relatedness estimates among different sets of the breeding population. Within-race relationship coefficients amongst founders (none of which were genotyped) in **H** matrices were greater than zero and provide further support for differentiation of races and population structure (Table 2). The Strzelecki Ranges population displayed the largest mean within-race relationship coefficient amongst founders (**H_JOINT_** 0.055; Table 2). Between-race or landrace relationships amongst founders in **H_JOINT_**varied significantly from zero in both directions, with 17 inter-race/landrace combinations significantly less than zero, 16 significantly greater than zero, and 12 not significantly different to zero (Table 3). Cryptic relationships among the native range populations provided evidence of population structure and evidence of a Tasmanian origin rather than the mainland or Bass Straight origin of third-party populations (Portugal and Californian landraces). The Furneaux race was significantly dissimilar to all other races/landraces apart from the nearby Northeast Tasmania, while Western Tasmania showed the greatest similarity to other races/landraces with five inter-race/landrace combinations significantly greater than zero (Table 3). Relationships amongst ungenotyped founders in **H_JOINT_** were around one-quarter the value of those amongst their genotyped OP offspring.

**Table 2:** Mean, standard deviation, minimum, and maximum within-race relationship coefficient in **H_JOINT_** for founders of each native range population and landrace

Race	Mean*^a^*	SD	Min	Max
Strzelecki ranges	0.055***	0.048	−0.038	0.253
Furneaux	0.007***	0.013	−0.025	0.104
NE Tasmania	0.011***	0.016	0.000	0.041
SE Tasmania	0.034***	0.037	−0.003	0.175
S Tasmania	0.138	0.000	0.138	0.138
W Tasmania	0.006**	0.007	0.000	0.017
King Island	0.021***	0.022	−0.012	0.073
W Otways	0.042***	0.036	−0.026	0.149
E Otways	0.012***	0.019	−0.008	0.063
Portugal	0.045***	0.067	−0.224	0.328
California	0.006	0.022	−0.024	0.041

a One-way *t*-test result for *H_1_*: mean significantly greater than zero: ^*^P < 0.05, ^**^P < 0.01, ^***^P < 0.001.

**Table 3:** Mean inter-race relationship coefficient amongst founders in H_JOINT_

Race*^a^*	STRZ	FURNX	NE_TAS	SE_TAS	W_TAS	KI	W_OTW	E_OTW	PORT
FURNX	−0.008***								
NE_TAS	−0.002*	0.002**							
SE_TAS	−0.002*	−0.003***	0.017***						
W_TAS	0.001	−0.001**	0.003	0.007***					
KI	0.000	−0.005***	−0.008**	0.000	0.010***				
W_OTW	0.019***	−0.006***	−0.004**	−0.004**	0.005***	0.023***			
E_OTW	0.014***	−0.003***	*−*0.001	0.000	0.000	0.005***	0.018***		
PORT	−0.006***	−0.006***	0.014***	0.039***	0.008***	*−*0.002	−0.004**	0.000	
CALIF	−0.007***	−0.006***	0.007	0.023***	0.003*	0.006	−0.007***	*−*0.003	0.025***

Means significantly less than zero shown in italics. Means significantly greater than zero shown in bold type.^b^

a STRZ: Strzelecki Ranges; FURNX: Furneaux; NE_TAS: NE Tasmania; SE_TAS: SE Tasmania; W_TAS: W Tasmania; KI: King Island; W_OTW: W Otways; E_OTW: E Otways; PORT: Portugal; CALIF: California.

b Two-way *t*-test results for *H_1_*: mean significantly different to zero: ^*^P < 0.05, ^**^P < 0.01, ^***^P < 0.001.

### Genetic parameter estimates from traditional ABLUP models and HBLUP models

While there are consistent increases in additive variance estimates when moving from ABLUP_+race_ to both ABLUP_−race_ and HBLUP, the changes in heritability, dominance variance, and genetic correlations are similar to the standard error estimates. These populations do not provide strong evidence that genetic parameter estimates derived from ABLUP differ dramatically from estimates derived from HBLUP models.

As expected, removing the race/landrace genetic groups from the EG2 pedigree caused significant increases in ${\hat{CV}}_{a}$ and ${\hat{h}}^{2}$ from individual-site analyses of VOL, while ${\hat{d}}^{2}$ was not significantly affected (Table 4). The removal of fixed race effects resulted in consistently poorer model fit to single-site VOL data [Akaike information criteria (AIC) on average 41.9 higher; Table 4]. HBLUP models fit the single-site VOL data marginally better than the ABLUP_−race_ models (AIC on average was 2.3 lower). The ${\hat{CV}}_{a}$ and ${\hat{h}}^{2}$ of HBLUP models were intermediate between respective values for ABLUP models that included and excluded race (Table 4) providing a reduction in bias from excluding population structure.

**Table 4:** Mean and standard error of Akaike information criterion (AIC), coefficient of additive variation (${\hat{CV}}_{a}$), narrow-sense heritability (${\hat{h}}^{2}$), and dominance proportion (${\hat{d}}^{2}$) for stem volume at single sites analysed by ABLUP with races included as genetic groups (ABLUP_+race_), ABLUP without groups (ABLUP_-race_), and HBLUP without groups

	ABLUP_+race_	ABLUP_-race_	HBLUP	Δrace*^a^*	Δrace +markers*^b^*
AIC	21,209	21,251	21,248	41.9 (2.6)***	39.6 (2.4)***
${\hat{CV}}_{a}$	14.4 (0.9)	15.8 (0.9)	15.5 (0.8)	1.4 (0.5)**	1.1 (0.5)*
${\hat{h}}^{2}$	0.139 (0.017)	0.160 (0.018)	0.153 (0.017)	0.021 (0.007)**	0.014 (0.006)*
${\hat{d}}^{2}$	0.144 (0.016)	0.141 (0.016)	0.139 (0.016)	*−*0.003 (0.002)	*−*0.005 (0.002)*

All 18 individual site estimates are from the EG2 program, which provided trial data from all three regions.

a Significance of two-sided tests of significance for change in estimates when genetic groups are removed from model, and

b when the H matrix is used; ^*^P < 0.05, ^**^P < 0.01.

Similar patterns were observed for the EG2 cross-site analyses of VOL. AIC indicated poorer model fit (115.2 greater) for the ABLUP model when race effects were removed from the pedigree, which was slightly improved by using HBLUP instead of ABLUP_−race_ (AIC difference of 13.5; Table 5). Removing race effects led to a similar decline in model fit for the cross-site EG1 VOL analysis (AIC 256.8 larger; Table 5). However, the HBLUP model fit the EG1 cross-site VOL data better than the benchmark model of ABLUP_+Race_ (AIC 26.5 lower; Table 5). The reason for a distinctively better model fit using HBLUP on EG1 data is unknown—it could be due to the greater number of genotyped trees in the EG1 program or different patterns of parent-within-race structure between the programs.

**Table 5:** Estimates of AIC, additive variance (${\hat{\sigma }}_{a}^{2}$), heritability (${\hat{h}}^{2}$), and dominance proportion (${\hat{d}}^{2}$) for each region, and between region additive genetic correlations (${\hat{r}}_{a})$ for different multisite models within each program and across programs jointly for stem volume

Program:	EG1					EG2					JOINT
Model:	ABL UP_+race_*^a^*	ABL UP_−race_*^b^*	HBL UP*^c^*	Δrace*^d^*	Δrace +markers*^e^*	ABL UP_+race_*^a^*	ABL UP_−race_*^b^*	HBL UP*^c^*	Δrace*^d^*	Δrace +markers*^e^*	HBLUP
AIC	245,132.7	245,389.5	245,106.2	256.8	*−*26.5	156,003.3	156,118.5	156,105.0	115.2	101.7	*−*
${\hat{\sigma }}_{a(WA)}^{2}$	0.523 (0.045)	0.611 (0.046)	0.598 (0.046)	0.088	0.075	1.035 (0.252)	1.420 (0.298)	1.353 (0.289)	0.385	0.318	0.651 (0.047)
${\hat{\sigma }}_{a(GT)}^{2}$	0.825 (0.141)	1.010 (0.150)	1.009 (0.151)	0.185	0.184	1.038 (0.179)	1.242 (0.198)	1.219 (0.195)	0.382	0.181	0.883 (0.100)
${\hat{\sigma }}_{a(\mathrm{GIPPS})}^{2}$	NA	NA	NA	NA	NA	0.882 (0.191)	0.975 (0.195)	1.113 (0.218)	0.093	0.231	0.509 (0.111)
${\hat{h}}_{WA}^{2}$	0.11 (0.01)	0.12 (0.01)	0.13 (0.01)	0.01	0.02	0.08 (0.02)	0.10 (0.02)	0.10 (0.02)	0.02	0.02	0.14 (0.01)
${\hat{h}}_{GT}^{2}$	0.09 (0.01)	0.08 (0.01)	0.11 (0.02)	0.01	0.02	0.17 (0.03)	0.20 (0.03)	0.20 (0.03)	0.03	0.03	0.12 (0.02)
${\hat{h}}_{\mathrm{GIPPS}}^{2}$	NA	NA	NA	NA	NA	0.08 (0.02)	0.09 (0.02)	0.10 (0.02)	0.01	0.02	0.05 (0.01)
${\hat{d}}_{WA}^{2}$	0.13 (0.01)	0.13 (0.01)	0.13 (0.01)	0.00	0.00	0.05 (0.01)	0.05 (0.01)	0.05 (0.01)	0.00	0.00	0.14 (0.01)
${\hat{d}}_{GT}^{2}$	0.06 (0.01)	0.06 (0.01)	0.06 (0.01)	0.00	0.00	0.12 (0.01)	0.12 (0.01)	0.12 (0.01)	0.00	0.00	0.10 (0.02)
${\hat{d}}_{\mathrm{GIPPS}}^{2}$	NA	NA	NA	NA	NA	0.07 (0.01)	0.07 (0.01)	0.07 (0.01)	0.00	0.00	0.12 (0.02)
${\hat{r}}_{a(WA,GT)}$	0.59 (0.08)	0.65 (0.06)	0.63 (0.07)	0.06	0.04	0.82 (0.07)	0.83 (0.06)	0.84 (0.05)	0.01	0.02	0.75 (0.05)
${\hat{r}}_{a(WA,\mathrm{GIPPS})}$	NA	NA	NA	NA	NA	0.78 (0.09)	0.84 (0.06)	0.82 (0.07)	0.06	0.04	0.83 (0.08)
${\hat{r}}_{a(GT,\mathrm{GIPPS})}$	NA	NA	NA	NA	NA	0.78 (0.06)	0.80 (0.05)	0.82 (0.05)	0.02	0.04	0.69 (0.08)

Standard errors of estimates are in parentheses.

a ABLUP_+−race_ derived from pedigree with genetic groups.

b ABLUP_−race_ derived from pedigree without genetic groups.

c HBLUP derived from pedigree and GRM.

d Δrace is change in estimate when genetic groups are removed from the model.

e Δrace + markers is change when genetic groups are removed from the model and the H matrix is used.

Removal of race effects from the ABLUP model reallocated variance from fixed genetic groups to variance among parents, inflating ${\hat{\sigma }}_{a}^{2}$ and the derived ${\hat{h}}^{2}$ estimates. The ${\hat{\sigma }}_{a}^{2}$ for VOL increased by an average of 21% across regions and programs (Table 5) when race effects were excluded from the model. Some of these increases were reduced when progressing to the cross-site HBLUP models for each program, although ${\hat{\sigma }}_{a}^{2}$ and ${\hat{h}}^{2}$ remained significantly larger than ABLUP_+race_ models (Table 5). HBLUP cross-site ${\hat{h}}^{2}$ estimates for VOL were 0.13 ± 0.01 and 0.11 ± 0.02 in the EG1 program in WA and GT, respectively, and 0.10 ± 0.02, 0.20 ± 0.03, and 0.10 ± 0.02 in the EG2 program in WA, GT, and GIPPS, respectively. The joint-program HBLUP produced ${\hat{h}}^{2}$ estimates for VOL of 0.14 ± 0.01, 0.12 ± 0.02, and 0.05 ± 0.01 in WA, GT, and GIPPS, respectively (Table 5). Estimates of ${\hat{d}}^{2}$ for VOL were not affected by choice of NRM and estimates from the joint analysis were 0.14 ± 0.01, 0.10 ± 0.02, and 0.12 ± 0.02 in WA, GT, and GIPPS, respectively (Table 5). Additive genetic correlations amongst regions were slightly increased by the removal of race effects and ranged from 0.69 ± 0.08 for ${\hat{r}}_{a(\mathrm{GT},\mathrm{GIPPS})}$ to 0.83 ± 0.08 for ${\hat{r}}_{a(\mathrm{WA},\mathrm{GIPPS})}$ from the joint HBLUP model (Table 5). Inter-region dominance correlation estimates from joint HBLUP were 0.31 for ${\hat{r}}_{d(\mathrm{WA},\mathrm{GT})}$, 0.10 for ${\hat{r}}_{d(\mathrm{WA},\mathrm{GIPPS})}$, and 0.62 for ${\hat{r}}_{d(\mathrm{GT},\mathrm{GIPPS})}$ (not tabulated).

Estimated heritability in DENS was greater in the EG2 program (0.58 ± 0.08) based on core samples than the EG1 program (0.28 ± 0.03) based on penetrometer data, with an intermediate ${\hat{h}}_{\mathrm{Xsite}}^{2}$ of 0.34 ± 0.02 from the joint analysis (Table 6). The programs each displayed cross-site ${\hat{h}}_{\mathrm{Xsite}}^{2}$ around 0.30 for CELL, with a joint estimate of 0.29 ± 0.03 (Table 6). Dominance effects were significant for both wood quality traits with ${\hat{d}}_{\mathrm{Xsite}}^{2}$ of 0.10 from joint HBLUP analysis.

**Table 6:** Summary of variance functions from multisite HBLUP models fitted to outer-wood density (DENS) and cellulose content (CELL) for each program separately and jointly

	DENS	CELL
*EG1*		
${\hat{h}}_{\mathrm{Xsite}}^{2}$	0.28 (0.03)	0.30 (0.04)
${\hat{d}}_{\mathrm{Xsite}}^{2}$	0.13 (0.02)	0.08 (0.02)

*EG2*		
${\hat{h}}_{\mathrm{Xsite}}^{2}$	0.58 (0.08)	0.31 (0.07)
${\hat{d}}_{\mathrm{Xsite}}^{2}$	0.01 (0.06)	0.09 (0.05)

*JOINT*		
${\hat{h}}_{\mathrm{Xsite}}^{2}$	0.34 (0.02)	0.29 (0.03)
${\hat{d}}_{\mathrm{Xsite}}^{2}$	0.10 (0.02)	0.10 (0.02)

### Breeding value accuracy from traditional ABLUP models and HBLUP models

**Figure 4:**
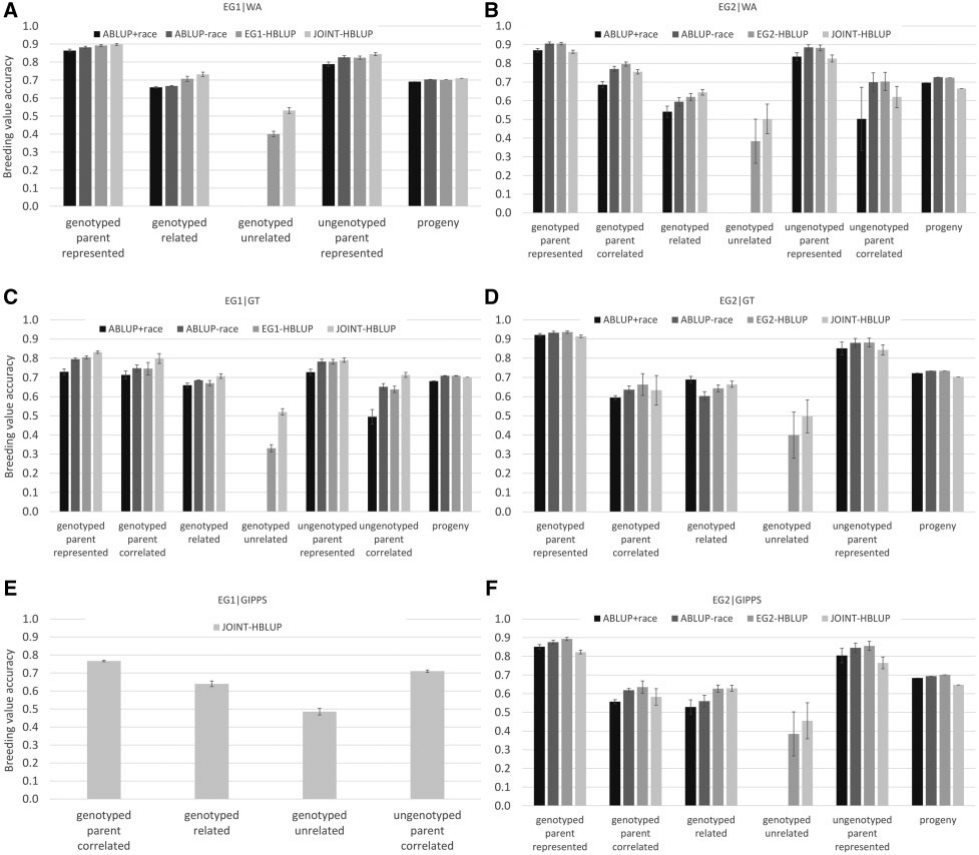
Mean breeding value accuracy for different classes of individuals estimated by ABLUP models with and without fixed race effects, within-program HBLUP, and joint HBLUP for (A) EG1 individuals in WA, (B) EG2 individuals in WA, (C) EG1 individuals in GT, (D) EG2 individuals in GT, (E) EG1 individuals in GIPPS, and (F) EG2 individuals in GIPPS. Error bars are standard errors of means.

Overall, the greatest breeding value accuracy estimates (${\hat{r}}_{g\hat{g}}$) were observed for genotyped parents represented in the prediction region, followed by ungenotyped parents represented in the prediction region (Figure 4 and see Supplementary Tables S2, A and B for more details). HBLUP models produced smaller PEV estimates than the benchmark ABLUP_+race_ models for the same parents (see Supplementary Table S2), resulting in generally higher estimates of ${\hat{r}}_{g\hat{g}}$. An exception was for EG2 parents, where even though PEVs were significantly smaller, ${\hat{r}}_{g\hat{g}}$ from joint HBLUP were similar to ${\hat{r}}_{g\hat{g}}$ from ABLUP_+race_ due to the substantially smaller ${\hat{\sigma }}_{a}^{2}$ of the joint HBLUP model (see Table 5).

Prediction accuracy for parents of progeny tested in other regions improved markedly in the joint HBLUP analyses (“parents correlated”; Figure 4). For EG1 parents of this class in GT, accuracy increased by 12% above the ABLUP_+race_ reference value (0.086 ± 0.023 above 0.713) when parents were genotyped and accuracy increased by 44% (0.218 ± 0.029) when they were not genotyped. For EG2 parents that were unrepresented by progeny in WA, ${\hat{r}}_{g\hat{g}}$ increased by 10% (0.070 ± 0.011) when parents were genotyped and by 23% (0.117 ± 0.114) when they were not genotyped (Figure 4 and Supplementary Table S2). Accuracy estimates of genotyped individuals not represented as parents but related through the pedigree (“genotyped related”; Figure 4) were also generally greater when genotype and pedigree were blended. In this class, joint HBLUP produced improvements of 11% and 7% (0.072 ± 0.011 and 0.048 ± 0.017) in WA and GT for EG1, respectively, and 19% for both GIPPS and WA (0.100 ± 0.036 and 0.104 ± 0.029, respectively) for EG2 (Figure 4 and Supplementary Table S2). Genotyped individuals unrelated to parents through the pedigree were included in HBLUP models, and their breeding values were estimated with an accuracy ${\hat{r}}_{g\hat{g}}$ of approximately 0.5 from the joint model (Figure 4).

Accuracy of progeny EBVs was the least impacted by choice of relationship matrix. Average PEVs for progeny were marginally higher for EG1 (by 0.050 and 0.008 SD_a_ units in WA and GT, respectively, under the joint HBLUP model), although average ${\hat{r}}_{g\hat{g}}$ was increased by 3% (0.019 and 0.020) in WA and GT, respectively, due to larger values of ${\hat{\sigma }}_{a}^{2}$ (Figure 4, Table 5, and Supplementary Table S2). Average PEVs for progeny were 37%, 32%, and 10% smaller (by 0.173, 0.169, and 0.051 SD_a_ units) for EG2 in GIPPS, WA, and GT, respectively, when estimated with the joint HBLUP model (Supplementary Table S2) . However, average accuracy from joint HBLUP was 3–5% lower for EG2 progeny across regions due to the lower values of ${\hat{\sigma }}_{a}^{2}$ relative to the EG2 cross-site models (Figure 4, Table 5, and Supplementary Table S2).

## Discussion

The single-step GBLUP (HBLUP) approach was used to integrate genomic, pedigree, and phenotypic information and provide unbiased predictions of genetic merit for breeding programs with few pedigree connections. Genotyping the parents that were evaluated in separate progeny trial networks created sufficient connectivity between the programs to provide predictions of genetic merit for parents in regions where they were not evaluated and also improved the accuracy of predictions. Although the benefits of HBLUP to tree improvement have been demonstrated in a number of studies (Cappa *et al.* 2017; Ratcliffe *et al.* 2017; Klápště *et al.* 2018, 2020; Cappa *et al.* 2019; Thavamanikumar *et al.* 2020; Ukrainetz and Mansfield 2020), this is the first published report of an HBLUP application that connects large, independent, multigenerational tree breeding programs so that breeding value predictions may be directly compared and inferred in regions where little to no testing has been undertaken.

A clear advantage of HBLUP is that relationships amongst genotyped individuals are quantified precisely, rather than represented by the expected average relationship derived from the pedigree. This advance has been well documented in tree breeding contexts (Munoz *et al.* 2014; Gamal El-Dien *et al.* 2016; Ratcliffe *et al.* 2017; Klápště *et al.* 2018) and implied that genotyping may be used to discover errors in the pedigrees, reveal unknown connections within pedigrees, as well as to improve the precision of relationship estimates. While the transfer of relationship information from genotyped to ungenotyped individuals is also well established (Legarra *et al.* 2009; Aguilar *et al.* 2010; Ratcliffe *et al.* 2017; Thavamanikumar *et al.* 2020), our method of genotyping progeny tested parents from large breeding populations provides a novel approach to utilizing cryptic relationships amongst population founders. The approach provides breeding programs with information to guide the exchange of germplasm as well as predictions of how individual trees with genotype data will perform in regions where the breeding program has not established trials. It also provides a more powerful platform for quantifying reaction norms to understand which climate and soil variables lead to changes in breeding value predictions and where specific parents should be deployed.

Positive associations amongst founders within races are expected due to coevolution and estimates of genetic distance among races implicit in **G** are incorporated into **H**. The true average relationship coefficient amongst randomly selected individuals within races is expected to be substantially larger than the estimates in the **H** matrices and shown in Table 2. Evidence supporting this comes from observing that relationships in **H** amongst ungenotyped founders that were unrelated though the pedigree were typically around 25% of those directly estimated in **G** amongst their OP offspring. Relationships in **G** and **H** are based on the probability that alleles are identical by state rather than by descent, and moreover, the adjustment in **H** made by projecting **G** onto relationships in **A**_11_ is relatively small as determined by the coefficient $A_{12}A_{22}^{-1}$ in Equation 7.

Further evidence that relationships amongst founders were underestimated in **H** is that HBLUP models did not fully correct the inflation in ${\hat{\sigma }}_{a}^{2}$ and ${\hat{h}}^{2}$ that was observed by removing fixed-effect race groups from the pedigree. This may alternatively indicate that the inclusion of genetic groups provides an overly strong assumption of within-group relatedness. The practice of fitting races or provenances as genetic groups (Quaas 1988; Westell *et al.* 1988) is well established in tree breeding (examined by Dutkowski *et al.* 1997) and ensures that heritable genetic variation is estimated at the within-race or within-provenance level. When genetic groups were removed from the pedigree, we observed the expected increases in ${\hat{\sigma }}_{a}^{2}$ and ${\hat{h}}^{2}$ in ABLUP models, as genetic variance that had been apportioned to race effects was pooled with ${\hat{\sigma }}_{a}^{2}$. ABLUP models that excluded genetic group effects provided a poorer fit as indicated by larger AIC estimates. The greater prediction accuracy associated with ABLUP models that excluded genetic groups is therefore associated with the inflation of genetic variance and does not indicate an improvement in overall model fit.

Progression from ABLUP_−race_ to HBLUP models moderated the inflation of $\sigma_{a}^{2}$ estimates for both populations, using both single-site and more complex multisite models. It is expected that ${\hat{\sigma }}_{a}^{2}$ and ${\hat{h}}^{2}$ will be more similar between HBLUP and ABLUP_+race_ models when within-race relationships are correctly accounted for with **H** (Lourenco *et al.* 2020). Alternatives for incorporating groups in HBLUP are currently being explored (Bradford *et al.* 2019) to reduce the bias of genomic predictions without reducing accuracy (Garcia-Baccino *et al.* 2017). Options include following the approach of Quaas (1988) and Westell *et al.* (1988) to represent groups in **A** or **H** (Misztal *et al.* 2013b) and treating groups as “metafounders,” which are quasi-individuals with inbreeding values representing the degree of similarity within the group and relationship coefficients representing associations with other metafounders (Legarra *et al.* 2015). Extending HBLUP models to accommodate ancestral race effects is a priority for forest tree breeding populations that are often derived from diverse wild populations.

Genetic parameter estimates are similar to those previous published for *E. globulus*. Araujo *et al.* (2012) pooled data from 40 sites and reported heritability (${\hat{h}}^{2}$) and dominance variance estimates (${\hat{d}}^{2}$) of 0.11 ± 0.03 and 0.09 ± 0.02, respectively, while Costa e Silva *et al.* (2004) pooled data from five sites and reported ${\hat{h}}^{2}$ and ${\hat{d}}^{2}$ of 0.10 ± 0.04 and 0.04 ± 0.05, respectively for DBH in Portugal. These results are generally comparable with our joint HBLUP results for VOL, although the ${\hat{d}}^{2}$ estimates from these trials are greater (see Table 5). Cross-site pooled analyses of DENS have not been previously reported for *E. globulus*, so average single-site results are referenced for comparisons. Costa e Silva *et al.* (2009) reported an average ${\hat{h}}^{2}$ of 0.29 for Pilodyn penetration across eight Portuguese sites and Li *et al.* (2007) reported average ${\hat{h}}^{2}$ of 0.44 for DENS across three Australian sites. Our joint HBLUP analysis across 20 sites produced an intermediate ${\hat{h}}^{2}$ (pooled estimate of 0.34 ± 0.02). Comparable univariate, cross-site, heritability estimates have not been published for full-sib families of *E. globulus* for cellulose content or pulp yield; however, half-sib families have yielded higher heritability estimates (*e.g.*, 0.40 ± 0.06 reported by Stackpole *et al.* 2010).

Our approach to modeling ${\hat{\sigma }}_{f}^{2}$ across sites represents a pragmatic treatment of specific combining ability (SCA) in the context of industrial breeding programs deploying CP seed. Although ${\hat{\sigma }}_{f}^{2}$ is large enough in populations of *E. globulus* evaluated as full-sib families to contribute toward improvements in growth, Araujo *et al.* (2012) reported an among-site or type-B correlation estimate for *E. globulus* family-specific effects of 0.41 ± 0.13, indicating significant re-ranking of families in 40 Portuguese trials. Our results provide divergent estimates of ${\hat{\sigma }}_{f}^{2}$ by site, which adds to the difficulty of utilizing SCA. The ${\hat{d}}^{2}$ estimates ranged between 0.10 and 0.14 for VOL at 34 of the 48 sites evaluated (“Group 2”). This provides motivation for continuing to evaluate full-sib families to identify those with advantageous SCA for deployment.

Combining data from two breeding programs in southern Australia allowed for the estimation of inter-region genetic correlations that were unavailable or based on few trials in certain regions. For example, the program that was based in WA established many trials in that region and had no trials in GIPPS. The estimates of additive genetic correlations between these regions were made available by the incorporation of relationships among the parents of the disjunct breeding programs. Inter-region genetic correlation estimates are slightly larger than those presented by Dutkowski *et al.* (2015) for Tree Breeding Australia’s *E. globulus* program (0.75 v. 0.58 for ${\hat{r}}_{a(\mathrm{WA},\mathrm{GT})}$, 0.83 v. 0.80 for ${\hat{r}}_{a(\mathrm{WA},\mathrm{GIPPS})}$, and 0.69 v. 0.49 for ${\hat{r}}_{a(\mathrm{GT},\mathrm{GIPPS})}$). This supports the continuation of research to understand the factors underlying the significant genotype by environment interactions evident at the regional scale in Australia.

One reason to recommend HBLUP to tree breeders is that most of the traditional mixed-model analysis approaches applied to traditional genetic analyses may be retained. Once the **H^−1^** matrix is constructed, it can be read directly into software such as ASReml and BLUPF90 with subsequent analyses conducted with all the power and flexibility of the mixed-model platform. HBLUP is therefore applicable to any trait and it has been demonstrated, for example, through analysis of Dothistroma needle blight on clonally replicated full-sib families of *Pinus radiata* (Klápště *et al.* 2020) and for blight resistance in American chestnut back-cross populations (Westbrook *et al.* 2020). Although we demonstrated the approach here by genotyping a mere 382 individuals, it is applicable to far larger genotyped numbers using the same methodology. For example, Tsuruta *et al.* (2021) recently conducted an HBLUP analysis with 2.3 M genotyped individuals and a complete pedigree of 13.6 M Holsteins.

Our results illustrate that HBLUP conferred little benefit to the prediction accuracy of parents and progeny within each program, possibly due to the very low proportion of genotyped individuals. PEV and ${\hat{r}}_{g\hat{g}}$ for parents and progeny were generally similar between ABLUP_−race_ and within-program HBLUP. On the other hand, PEV values for parents were generally reduced and accuracies improved by progressing from within-program HBLUP to joint HBLUP (see Supplementary Table S2), which generally resulted in larger values of ${\hat{r}}_{g\hat{g}}$ for parents. Parent EBVs produced by HBLUP and ABLUP were well correlated (results not presented). It could be argued that HBLUP would produce more informed breeding value predictions due to the greater precision in relationship definitions provided by **H** compared with **A**, and this is a subject of ongoing exploration and validation.

HBLUP offers a clear advantage over ABLUP for predicting the value of genotyped individuals with no phenotype data and no pedigree linkages with individuals evaluated in trial networks. Breeding values were estimated for 52 EG1 individuals and 5 EG2 individuals that were genotyped but had no connections via pedigree. While accuracy estimates were lower than parental estimates, at around 0.4 and 0.5 for within-program and joint HBLUP, respectively, indirect estimates of breeding values were produced. Joint HBLUP enabled the prediction of a complete set of EBVs for EG1 parents, genotyped individuals with no phenotype data, and progeny in the GIPPS region where no progeny trials were established. Extending genomic prediction models to connect distinct pedigrees allows breeding programs to leverage information from one another and infer performance in environments where populations have not been evaluated. This study provides empirical evidence that may be used to promote collaboration among tree improvement programs to better characterize the genetic merit of individuals in environments where they have not been evaluated. The need for breeding value predictions in untested environments is expected to increase as forestry organizations examine options for adapting to climate change (Pinkard *et al.* 2015; Keskitalo *et al.* 2016), including breeding for altered climatic conditions (Gray *et al.* 2016). The strength of prediction across environments is dependent not only on joint genomic information but also on the precision of environmental definition and the treatment of GxE in modeling.

## Conclusions

Using genotype data to blend disconnected pedigrees and phenotype data from separate breeding programs into a unified analysis produces unbiased breeding values for direct comparisons between programs and indirect predictions of merit in environments where individuals may not have been evaluated. The joint HBLUP analysis significantly improved prediction error variance of parents and genotyped individuals, provided similar estimates of genetic parameters, more accurate EBV predictions, and offered the profound advantage of EBV prediction for genotyped individuals in regions they had not been evaluated.

Overall, the genotyping proved useful for correcting pedigree errors, more precisely defining relationships within and among populations, identifying the source of landrace populations, and integrating genotyped individuals with no phenotype or pedigree connections into the prediction framework. Understanding the impacts of incorporating genetic groups in the estimation of **H** will further align the traditional genetic evaluation pipelines that underpin tree breeding programs with approaches that incorporate marker-derived relationships into prediction models.

## Data Availability

The genomic, pedigree, and phenotypic data were submitted through the GSA journals figshare portal: https://doi.org/10.25387/g3.14877189.
